# Clinical Features of Aberrations Chromosome 22q: A Pilot Study

**DOI:** 10.1055/s-0041-1739496

**Published:** 2021-11-09

**Authors:** Emine Ikbal Atli, Engin Atli, Sinem Yalcintepe, Selma Demir, Cisem Mail, Damla Eker, Yasemin Ozen, Hakan Gurkan

**Affiliations:** 1 Department of Medical Genetics, Faculty of Medicine, Trakya University, Edirne, Turkey

**Keywords:** 22q deletions, 22q duplications, Array CGH

## Abstract

**Objective** A significant number of genetic variations have been identified in chromosome 22, using molecular genetic techniques. Various genomic disorders on chromosome 22, including cat's eye syndrome caused by extra copies of the proximal region of the 22q chromosome, are now well-defined. Our aim in the study was to show phenotypic variability associated with rearrangements of the 22q chromosomal region.

**Methods** We focused our study on clinical aspects of these disorders, including genetic testing, genotype-phenotype correlation, and potential treatments. A total of 998 patients were referred for genetic analysis (Karyotyping, MLPA, array-CGH) during January 2015 to February 2020 because of intellectual deficiency, behavior issues, and/or multiple congenital abnormalities in several genetics departments. Informed consent was obtained from all the patients and/or their parents.

**Results** 22q11.21 or 22q13.33 microdeletions and 22q11.22-q11.23 microduplication were identified in 31 patients out of referrals. The 22q aberrations were detected in 31/998 patients, giving a prevalence of 3.1%. In this study, 18 patients with 22q11.2 (LCR22A-H) deletion, three patients with 22q13.31 deletion, 9 patients with 22q11.2 duplication and one patient with 22q13.31 duplication were identified. We report on the clinical and molecular characterization of 31 individuals with distal deletions and duplications of chromosome 22q.

**Conclusions** The current study demonstrated in the largest postnatal case series reporting the whole spectrum of atypical phenotypic and genotypic variations at 22q. We believe that when all the phenotypic differences are taken into account, various anomalies including developmental delay and intellectual disability might be considered as an indication to search for aberrations of 22q along with congenital heart diseases.

## Introduction

Chromosome 22 is the second smallest human chromosome, covering 1.6 to 1.8% of the human genome. The short arm (22p) of this acrocentric chromosome contains ribosomal genes, while the long arm (22q) contains genes encoding protein, and it is this region that is sequenced. Human chromosome 22 is an acrocentric chromosome spanning approximately 51 million base pairs, with more than 855 annotated genes. 1 2 A significant number of genetic variations have been identified in chromosome 22, using molecular genetic techniques. Nonmosaic trisomy and monosomy of chromosome 22 are observed in the prenatal period. Trisomy 22 is the second aneuploidy after trisomy 16 in spontaneous abortions, seen in approximately 2 to 5% of cases. 3 While most of these disorders are rare, the prevalence of some, such as velocardiofacial syndrome (also known as VCFS, 22q11 deletion syndrome, and DiGeorge syndrome [DGS]), is as common as 1: 2000 people. Various genomic disorders on chromosome 22, including cat's eye syndrome caused by extra copies of the proximal region of the 22q chromosome, are now well-defined ( Table 1 ). Cat's eye syndrome caused by extra copies of the proximal region of the 22q chromosome, VCFS, 22q11.2 duplication syndrome, 22q11.2 deletion syndrome, supernumerary der (22) t (11; 22), 3: 1 caused by malsegregation of the t (11; 22) syndrome, and 22q13.3 deletion syndrome are better defined today ( Table 1 ). 4 In addition to the genomic disorders with molecular causes listed in Table 1 , there are hundreds of disorders associated with a gene, but the underlying cause is unknown or the disturbances are determined by statistical methods. Pathogenic copy number variants (CNVs) and CNVs of unknown clinical significance have been identified and added to the genetic variations to the complexity of human diseases. 5 The definition of the proximal region of chromosome 22q11.2 illustrates such progress.

**Table 1:** Recurrent genomic disorders on chromosome 22

Location	Condition	Gene/locus MIM number	Gene(s)/ locus	Detection rate by microarray
*22q11.1*	Cat eye syndrome	115470	Multiple	Precise detection rate unknown. The supernumerary marker chromosome is detectable by array CGH.
*22q11.21*	DiGeorge/Velocardiofacial/22q11.21 deletion syndrome	188400	TBX1	More than 95% have a detectable deletion.
*22q11.21*	22q11.21 duplication syndrome	608363	Multiple	Approximately 99% have a detectable duplication.
*22q11.2*	22q11.2 distal microdeletion syndrome	611867	Multiple	Approximately 99% have a detectable deletion.
*22q12.2*	Neurofibromatosis 2	101000	NF2	15–21% have a detectable deletion.
*22q12.3*	Walker–Warburg/muscular dystrophydystroglycanopathy A1	236670	LARGE	Deletions uncommon, recessive condition.
*22q13.3*	22q13.3 deletion syndrome	606232	SHANK3	Approximately 99% have a detectable deletion.
*22q13.3*	Metachromatic leukodystrophy/arylsulfatase A deficiency	250100	ARSA	Rare deletions. Recessive condition.

**Fig. 1:**
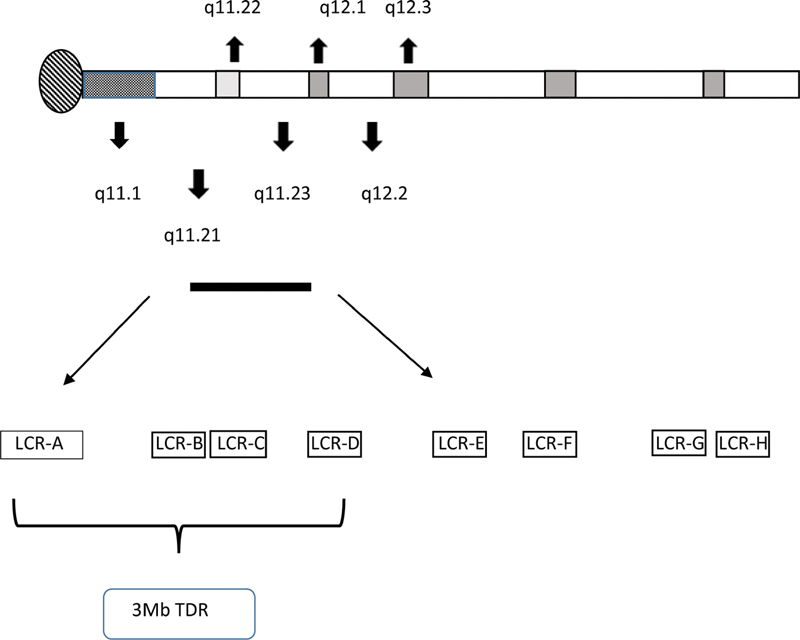
Schematic view of chromosome 22 indicating the position of the low-copy repeats in 22q11.2. LCR, low-copy repeat; TDR, typically deleted region.

This genomic region is rich in low copy repeats (LCRs), including LCR22A to LCR22H. Most of the genomic abnormalities of chromosome 22 occur as a result of LCR-mediated nonallelic homologous misalignments and intrachromosomal or interchromosome unequal recombination (NAHR) during meiosis. 6 In addition to the commonly deleted/duplicated region (LCRs A to D surrounded by LCR22s at both proximal and distal cutpoints for 3 Mb, 22q11.2 deletions and duplications), several “non-standard” deletions/duplication with variable breakpoints results from differential recombination from LCR22A to LCR22H ( Fig. 1 ). Some of these have been identified as separate deletion/duplication syndromes with varying genomic dimensions such as 22q11.2 distal deletion syndrome and 22q11.2 distal duplication syndrome. 7 8 In addition to the LCR-mediated NAHR mechanism, several other mechanisms have been proposed to explain the formation of CNVs in the human genome, including CNVs on chromosome 22 such as nonhomologous splicing, microhomology-mediated break-induced replication, and tandem repeats-mediated genomic rearrangement. Correct determination of the breakpoints of a CNV is a prerequisite for understanding the basic mechanisms that lead to its formation and establish CNV-phenotype correlation.

Our aim in the study was to show phenotypic variability associated with rearrangements of the 22q11.2 chromosomal region.

We focussed our study on clinical aspects of these disorders, including genetic testing, genotype-phenotype correlation, and potential treatments.

## Materials and Methods

All procedures performed in studies involving human participants were in accordance with the ethical standards of the institutional and/or national research committee (TUMF Scientific Research Ethics Committee Directive) and with the 1964 Helsinki Declaration and its later amendments or comparable ethical standards.

A total of 998 patients were referred for genetic analysis during January 2015 to February 2020 because of intellectual deficiency, behavior issues, and/or multiple congenital abnormalities in several genetics departments. Informed consent was obtained from all the patients and/or their parents. 22q11.21 or 22q13.33 microdeletions and 22q11.22-q11.23 microduplication were identified in 31 patients out of referrals. The clinical data of the 31 patients were retrospectively collected by a questionnaire sent to each referring clinician to obtain information on prenatal abnormalities, birth parameters, psychomotor development, neurological examination, behavioral pattern, growth, dysmorphism, associated malformations, paraclinical investigations, and familial history.

### Karyotype and FISH

Patients were referred for cytogenetic testing. Cytogenetic analysis on cultured blood lymphocytes was performed, according to standard protocols. Trypsin-Leishman G banded (GTL) metaphases were interpreted at a resolution level of 450 bands.

### MLPA

Multiplex ligation-dependent probe amplification was performed on DNA from peripheral blood lymphocytes. Kits P023B, P250 and P324-A1 for DGS/VCFS/CES (MRC-Holland, Amsterdam, Netherlands) were used. The three kits test 65 loci on 22q11 (8 on the CES region, 37 within the TDR and 20 adjacent distal to the TDR), 2 on 22q13, 7 on 4q, 1 on 7p, 5 on 8p, 2 on 9q, 9 on 10p, 1 on 10q, 6 on 17p, and 2 on 18q, most of them involved in the phenotypes of DGS and VCFS. Data analysis was made against up to five control samples using the MRC Coffalyser v8 and v9 softwares (MRC-Holland) or an in-house Excel spreadsheet.

Genomic DNA was extracted from the patients' peripheral lymphocytes using the QIAamp DNA Blood Midi kit (Qiagen, Valencia, CA). DNA concentration was determined with Nano- Drop ND-1000 spectrophotometer and software (NanoDrop Technologies, Berlin, Germany). Pathogenic CNVs were identified by CMA, using a 180 K CGH + SNP (ISCA design, Agilent, Santa Clara, CA) array, as part of the clinical testing for congenital anomalies, neurodevelopmental problems, and abnormal fetal ultrasound. Image was analyzed using CytoGenomics 2.7 software (Agilent Technologies, Santa Clara, CA, United States).

Validation of the microdeletion detected by array CGH was validated by fluorescence in situ hybridization. Fluorescence in situ hybridization (FISH) on metaphase spreads with probes for the DiGeorge critical region (D22S553, D22S942) as well as control probes for a more distal region on 22q13.3 (arylsulfatase A, LSI, ARSA, all from Vysis, Downers Groove, United States)

### Statistical Analysis

We performed one-tailed Chi-square tests and calculated *p* values at the 95% confidence level to determine the differences in proportion of observed cases between male and female patients. The proportion of cytogenetic abnormalities was expected to be equal (50% each) between males and females. Variables, presented as a percentage normalized to the total number of male or female patients, were compared. Findings with *p* < 0.05 values were accepted as statistically significant.

## Results

All patients had been referred for diagnostic testing by aCGH as part of standard clinical diagnostic workup. The 22q aberrations were detected in 31/998 patients, giving a prevalence of 3,1%. The patient cohort consisted predominantly of pediatric patients, with ages ranging from 5 days to 42 years, (mean= 9.9 years, median = 5 years). Although gender biases for other susceptibility CNVs such as 16p13.11 have been reported in different studies 9 , the sex ratio here was equivalent for patients (16 females, 15 males). Phenotypic details of the patients were ascertained first from the referral information provided prior to testing, and then by interrogation of patient notes and letters to the referring consultants, requesting detailed clinical information. Details of patients and clinical findings are provided in Table 2 .

**Table 2:** Molecular details and phenotypic features of individuals with 22q aberrations

Case	Abber.	chr22 band	bp start; stop (NCBI36/hg18)	Seg LCR22 A-H	Size	Origin	Age	Phenotypic features
1	Del	q11.21q11.22	18.894.835–21.440.514	C–D/E	2546kb	De novo	1	Loss of speech, developmental delay
2	Dup	q11.21q12.22	18.894.835–21.809.009	C–D/E	2,914.174kb	De novo	3	Kabuki makeup syndrome
3	Dup	q11.21q12.22	20.402.633–29.789.058	D-H	9386kb	Maternal	10	22q11.21 duplication
4	Dup	q11.21q12.22	20.719.112–29.789.058	D-H	9070kb	De novo	35	22q11.21 duplication?
5	Del	q13.31q13.33	45.988.776- 51.193.680		5205kb	De novo	1	Hypotonia
6	Del	q11.21q11.22	20.659.547–21.440.514	D-E	781kb	De novo	10	Mental retardation
7	Del	q11.21q11.22	18.729.944–21.505.417	B-E	2775kb	Maternal	5	speech disorder + rib cage deformity
8	Del	q11.21q11.22	19.036.998–21.505.417	D-E	2468kb	De novo	3	Postaxial polysyndactyly, hearing loss
9	Dup	q11.22-q11.23	23.258.229–23.648.163	H	390kb	De novo	31	History of four first trimester pregnancy loss
10	Dup	q13.31	48.360.180–50.151.126		1,790.947kb	De novo	3	Flattened nasal root, stuttering, craniosynostosis
11	Dup	q11.21q11.23	18.894.835–21.809.009	B-F	2,148.865kb	De novo	4	Dilated cardiomyopathy
12	Del	q11.2	18.894.835–21.505.417	B-E	2610kb	De novo	8	Developmental delay, intellectual disability
13	Del	q11.21q11.22	18.729.944–21.505.417	B-E	2775kb	De novo	27	Patients' 7 mother
14	Del	q11.2	18.729.944–21.440.514	B-E	2,710.571kb	De novo	1	Tetralogy of Fallot, growth retardation
15	Del	q11.2	22.313.381–22.556.733	F	243kb	De novo	5	Epilepsy, ADHD, fragile X
16	Del	q11.1-q11.21	17.084.955–19.659.894	A-C	2,574.94kb	De novo	13	Developmental delay, intellectual disability
17	Del	q11.21	20.719.112–21.440.514	E	721kb	De novo	13	Short stature, Williams syndrome
18	Del	q11.21	18.729.944–21.505.417	B-E	2,775.474kb	De novo	14	Tetralogy of Fallot, growth retardation, intellectual disability
19	Del	q11.21	18.628.019–21.440.514	B-E	2812kb	De novo	1	Operated ASD, VSD, aortic transposition
20	Del	q11.21	18.661.724–21.505.417	B-E	2,843.694kb	De novo	6	Mild intellectual disability, ADHD
21	Dup	q11.21	20.719.112–21.505.417	D-E	786,306kb	De novo	42	22q11.21 duplication
22	Dup	q11.21	18.661.724–21.809.009	B-F	3,147.286kb	De novo	5 months	Anal atresia, balanced translocation?
23	Dup	q11.1q11.23	16.133.474–21.505.417	A-E	5,371.944kb	De novo	27	Anal atresia, dysmorphic findings (+), hearing loss
24	Del	q11.1-q11.21	17.058.946–19.659.894	A-D	2,600.949	De novo	13	Speech and learning difficulties (twin sister of patient 16)
25	Del	q11.21	18.729.944–21.440.514	B-E	2,710.571kb	De novo	10	Operated tetralogy of Fallot, gait disturbance
26	Dup	q11.21	18.894.835–20.279.820	B-D	1,384.986kb	De novo	2	Strabismus, frontal bossing, flat nasal root, flat philtrum, thin lips, epilepsy
27	Del	q13.31-q13.33	21.440.514–51.224.252	E-…	6,670.17kb	De novo	2 months	Severe hypotonia
28	Del	q11.21	18.729.944–21.505.417	B-E	2,775.474kb	De novo	3 months	Pes equinovarus, anterior fontanelle closed, overlapping on toes
29	Del	q11.21	18.661.724–21.440.514	B-E	2.778.791kb	De novo	3	Neuromotor developmental delay
30	Del	q13.2-q13.33	43.572.90–51.224.252		7.651.344kb	De novo	1	Motor retardation
31	Del	q11.21	18.894.835–21.440.514	B-E	2,545.68kb	De novo	A month	Hypotonia, epilepsy, anteriorly located anus

Abbreviations: ADHD, attention deficit hyperactivity disorder; ASD, atrial septal defect; VSD, ventral septal defect.

In this study, 18 patients with 22q11.2 (LCR22A-H) deletion, three patients with 22q13.31 deletion, 9 patients with 22q11.2 duplication and one patient with 22q13.31 duplication were identified.

The phenotypes of the cases are summarized in Table 2 . The clinical phenotype varied among the individuals in this study, although a majority of cases displayed various degrees of developmental delay, ranging from mild to severe, and speech disturbances. Other clinical features present in more than five cases included behavioral problems, hypotonia, and dysmorphic facial features.

## Discussion

In the present study, we reported on the clinical and molecular characterization of 31 individuals with distal deletions and duplications of chromosome 22q. We detected 22q11.2 duplications in 9 patients and 22q13.31 duplications in one patient. Among the 998 patients tested, the estimated frequency of 22q11 and 22q13 duplications were approximately 1,002%. The estimated frequency in our patient population is slightly higher compared with the studies of Coppinger et al, who identified 18 distal duplications among 22,096 patients tested, and Wincent et al, who identified 16 distal duplications among 11,463 patients. Since a patient group with speech delay, brain malformations, and autism spectrum disorders was included in our study, this may have resulted from patient selection. 8 9 10 There is no standard procedure for screening for duplication in 22q11.2, because these patients show different clinical manifestations, only some of which are compatible with 22q11.2 deletion syndrome, usually in a milder phenotype. 11 12 13 14 Because the entire clinical spectrum is still unknown and most of these individuals overlap with normality, finding correlations of the sizes and positions of these duplications and phenotype is more difficult than deletions. 12 13

Deletions at 22q11.2 had a standard 3 Mb deletion in 87% of cases, a smaller, proximally nested 1.5 Mb deletion in 7% of cases, and other atypical deletions, nested, overlapping, or typically deleted region (TDR) clustered together. By NAHR after asynchronous replication, large low-copy repeats at 22q11,2 (LCR22s A to D) mediate repetitive deletions, while smaller LCRs (E-H) alongside recently described uncommon deletions or alternate breakpoints. 6 15 16 17 18

In this article, we present nine patients referred to us for genetic diagnosis of 22q11.2 deletion syndrome. We discuss screening diagnostic strategies for patients referred for the 22q11.2 deletion test as well as the clinical implications of these findings for a potential genotype-phenotype correlation.

The chromosome region 22q11.2 has long been recognized as a hotspot for genomic rearrangement and related disorders such as 22q11.2 deletion syndrome (DGS/VCFS, OMIM 188400/OMIM 192430), der(22) t(11; 22) syndrome (OMIM 609029), and cat-eye syndrome (OMIM 115470). Der(22) syndrome and cat eye syndrome are rare conditions characterized by an increased copy number of the most centromeric portion of 22q11, whereas 22q11.2 microdeletions are more common with an estimated frequency of 1 in 4,000 to 6,000 live births. 19 20

22q11 deletion syndrome is characterized by not only congenital heart defects, immunodeficiency, transient neonatal hypocalcemia, velopharyngeal insufficiency and a distinctive facial appearance but also learning disabilities and behavioral abnormalities. Phenotype varies with involvement of multiple organ systems. 5 21 22 The entire phenotypic spectrum of 22q11.2 deletion syndrome is provided by multiple dose-sensitive genes required for normal development across the 22q11.2 region. 23 Furthermore, nonoverlapping, atypical deletions have significantly overlapping phenotypes, suggesting several candidate genes for the syndrome, a common developmental pathway, or a positional gene effect at 22q11.2. 24 25 26 27 28 29 It has also been suggested that phenotypes represented by identical deletions at 22q11.2 are altered by parental imprinting, unbalanced regulatory effects, polymorphisms not masked by recessive mutations or hemizygosity, environmental factors, or stochastic events during morphogenesis. 29 30 31 32 Susceptibility to other syndromes has also been suggested in patients with the 22q11.2 deletion. 28 33 34 Several studies have identified several candidate genes at 22q11.2 that reproduce part of the phenotype or are suspected to alter disease susceptibility in animal models. 24 25 35 36 Accordingly, correlations made within a contiguous gene syndrome will always be subject to phenotype exclusions.

**Fig. 2:**
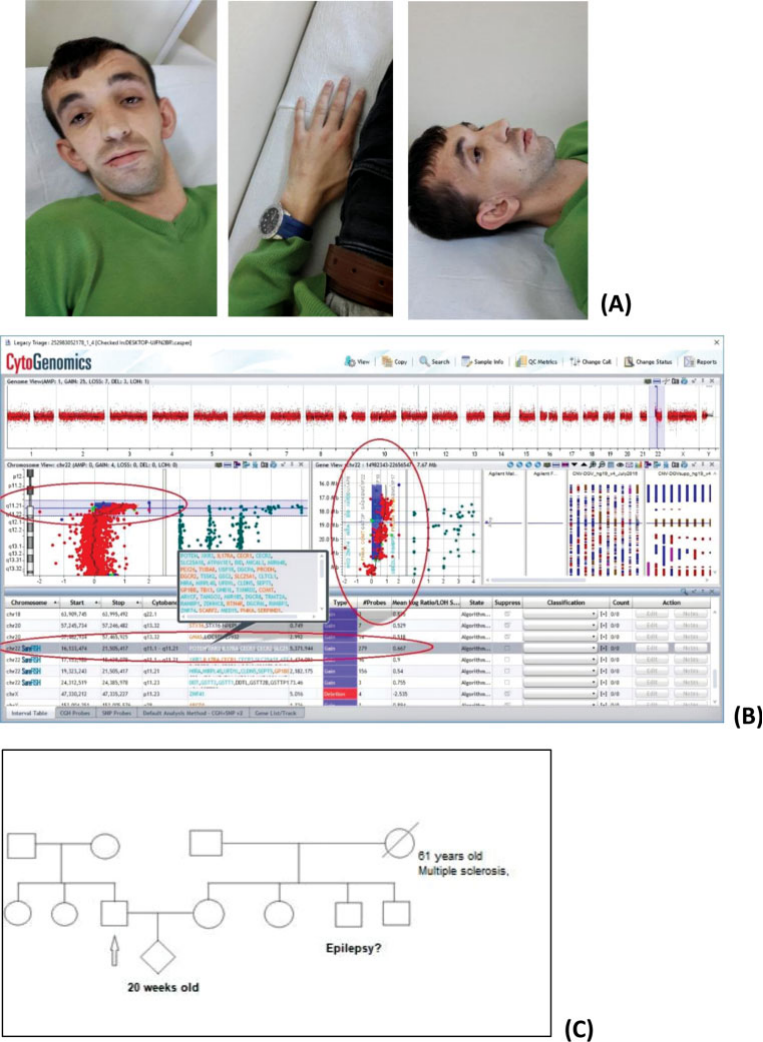
Photographs of patient no: 23 carrying an approximately 5.3 Mb 22q11.1q11.23 duplication ( **A** ), a-CGH image of case ( **B** ), pedigree of family ( **C** ).

In the present study, of the 18 patients carrying 22q11.2 (LCR22A–H) deletion, most of the deletions were spread in the region between LCRB and E. The clinical findings of patients with this deletion showed a wide spectrum ( Table 2 ). The reason for the wide phenotypic variation is unknown, but possible explanations may be that the 22q11.2 deletion syndrome phenotype may be due to other genetic mutations or that other genes not involved in duplication may compensate or hinder the pathogenesis of duplication. However, a milder phenotype was observed in 22q11 duplication carriers ( Fig. 2 ). Duplication of chromosome 22q11 is also characterized by highly variable features, which may range from normal to mild and may include intellectual disability or learning disabilities, growth retardation, hypotonia, and delayed psychomotor development. 37 38 The most frequently reported symptoms in the 22q11.2 duplication syndrome are mental retardation/learning difficulties (cognitive deficits such as deficits of memory performance, perceptual organization and verbal comprehension, ADHD and speech impairment) (97%), delayed psychomotor development (67%), growth retardation (63%), and muscular hypotonia (43%). The most common dysmorphic features detected are hypertelorism (70%), broad flat nose (53%), micrognathia (52%), velopharyngeal insufficiency (48%), dysplastic ears (45%), epicanthal folds (42%), and downslanting palpebral fissures (41%). Other reported symptoms are congenital heart malformation, visual and hearing impairment, seizures, microcephaly, ptosis, and urogenital abnormalities. 14

In our patient group, changes covering the 22q13 band region (3 deletions and 1 duplication) were detected in four of our patients ( Table 2 ). Clinical findings in our patients with deletion including 22q13.33 region are hypotonia and motor retardation. This region has been associated with Phelan–McDermid syndrome (PMS) (phenotype MIM number: 606232) in the literature. PMS is a developmental disorder with variable features. Common features include neonatal hypotonia, global developmental delay, normal to accelerated growth, absent to severely delayed speech, autistic behavior, and minor dysmorphic features. The responsible gene SHANK3 is located in this region. 39 40 41 Duplication was detected in 22q13.33 region in one of our patients. A 3-year-old patient had clinical findings of flattened nasal root, stuttering, and craniosynostosis ( Table 2 ). In duplication, it included the SHANK3 gene as in deletions of the same region. On the basis of the phenotype of SHANK3-overexpressing mice, Han et al hypothesized that SHANK3 overexpression may have a role in hyperkinetic neuropsychiatric disorders in humans. 42 Duplication of the 22q13 region is included in the literature as chromosome 22q13 duplication syndrome (phenotype MIM number: 615538).

A larger deletion was detected in the 22q13.2q13.33 region of approximately 7.5 Mb in one of our patients. Although the deletion of the patient corresponded to the PMS syndrome region, it was observed that it spreads beyond this area. For instance, neonatal hypotonia and late walking were reported in 20% of those with deletions of just 22q13.33, yet were reported for more than 90% of those with the largest deletions (22q13.2). The traditional approach would ignore the difference in frequency and identify 22q13.33 as being the only candidate region. 43 Isolated motor retardation was found in the clinical findings of our patient.

The current study demonstrated in the largest postnatal case series reporting the whole spectrum of atypical phenotypic and genotypic variations at 22q. The knowledge regarding the distribution of findings within and associated with various organ systems may enable a rapid and precise diagnostic process. We believe that when all the phenotypic differences are taken into account, various anomalies including developmental delay and intellectual disability might be considered as an indication to search for aberrations of 22q along with congenital heart diseases. All of this data will contribute to the establishment of the true prevalence of these anomalies and defects, and reveal the importance of the multidisciplinary counseling and the contribution of this condition to positive outcomes.

## References

[1] Cole C G McCann O T Collins J E Finishing the finished human chromosome 22 sequence Genome Biol 2008 9 05 R78 18477386 10.1186/gb-2008-9-5-r78 PMC2441464

[2] Dunham I Shimizu N Roe B A The DNA sequence of human chromosome 22 Nature 1999 402(6761):489 495 10591208 10.1038/990031

[3] Yu S Graf W D Shprintzen R J Genomic disorders on chromosome 22 Curr Opin Pediatr 2012 24 06 665 671 23111679 10.1097/MOP.0b013e328358acd0

[4] Robin N H Shprintzen R J Defining the clinical spectrum of deletion 22q11.2 J Pediatr 2005 147 01 90 96 16027702 10.1016/j.jpeds.2005.03.007

[5] Yu S Graf W D Ramalingam A Identification of copy number variants on human chromosome 22 in patients with a variety of clinical findings Cytogenet Genome Res 2011 134 04 260 268 21849782 10.1159/000330123

[6] Shaikh T H O'Connor R J Pierpont M E Low copy repeats mediate distal chromosome 22q11.2 deletions: sequence analysis predicts breakpoint mechanisms Genome Res 2007 17 04 482 491 17351135 10.1101/gr.5986507 PMC1832095

[7] Ben-Shachar S Ou Z Shaw C A 22q11.2 distal deletion: a recurrent genomic disorder distinct from DiGeorge syndrome and velocardiofacial syndrome Am J Hum Genet 2008 82 01 214 221 18179902 10.1016/j.ajhg.2007.09.014 PMC2253964

[8] Coppinger J McDonald-McGinn D Zackai E Identification of familial and de novo microduplications of 22q11.21-q11.23 distal to the 22q11.21 microdeletion syndrome region Hum Mol Genet 2009 18 08 1377 1383 19193630 10.1093/hmg/ddp042 PMC2664143

[9] Tropeano M Ahn J W Dobson R J Male-biased autosomal effect of 16p13.11 copy number variation in neurodevelopmental disorders PLoS One 2013 8 04 e61365 23637818 10.1371/journal.pone.0061365 PMC3630198

[10] Wincent J Bruno D L van Bon B W Sixteen new cases contributing to the characterization of patients with distal 22q11.2 microduplications Mol Syndromol 2010 1 05 246 254 22140377 10.1159/000327982 PMC3214948

[11] Ensenauer R E Adeyinka A Flynn H C Microduplication 22q11.2, an emerging syndrome: clinical, cytogenetic, and molecular analysis of thirteen patients Am J Hum Genet 2003 73 05 1027 1040 14526392 10.1086/378818 PMC1180483

[12] Courtens W Schramme I Laridon A Microduplication 22q11.2: a benign polymorphism or a syndrome with a very large clinical variability and reduced penetrance?–Report of two families Am J Med Genet A 2008 146A 06 758 763 18260141 10.1002/ajmg.a.31910

[13] Ou Z Berg J S Yonath H Microduplications of 22q11.2 are frequently inherited and are associated with variable phenotypes Genet Med 2008 10 04 267 277 18414210 10.1097/GIM.0b013e31816b64c2

[14] Wentzel C Fernström M Öhrner Y Annerén G Thuresson A C Clinical variability of the 22q11.2 duplication syndrome Eur J Med Genet 2008 51 06 501 510 18707033 10.1016/j.ejmg.2008.07.005

[15] Saitta S C Harris S E Gaeth A P Aberrant interchromosomal exchanges are the predominant cause of the 22q11.2 deletion Hum Mol Genet 2004 13 04 417 428 14681306 10.1093/hmg/ddh041 PMC2836129

[16] Shaikh T H Kurahashi H Saitta S C Chromosome 22-specific low copy repeats and the 22q11.2 deletion syndrome: genomic organization and deletion endpoint analysis Hum Mol Genet 2000 9 04 489 501 10699172 10.1093/hmg/9.4.489

[17] Baumer A Riegel M Schinzel A Non-random asynchronous replication at 22q11.2 favours unequal meiotic crossovers leading to the human 22q11.2 deletion J Med Genet 2004 41 06 413 420 15173225 10.1136/jmg.2003.016352 PMC1735820

[18] Kurahashi H Tsuda E Kohama R Another critical region for deletion of 22q11: a study of 100 patients Am J Med Genet 1997 72 02 180 185 9382139 10.1002/(sici)1096-8628(19971017)72:2<180::aid-ajmg10>3.0.co;2-j

[19] Botto L D May K Fernhoff P M A population-based study of the 22q11.2 deletion: phenotype, incidence, and contribution to major birth defects in the population Pediatrics 2003 112(Pt 1):101 107 12837874 10.1542/peds.112.1.101

[20] Yamagishi H The 22q11.2 deletion syndrome Keio J Med 2002 51 02 77 88 12125909 10.2302/kjm.51.77

[21] Shprintzen R J Goldberg R B Young D Wolford L The velo-cardio-facial syndrome: a clinical and genetic analysis Pediatrics 1981 67 02 167 172 7243439

[22] Scambler P J Kelly D Lindsay E Velo-cardio-facial syndrome associated with chromosome 22 deletions encompassing the DiGeorge locus Lancet 1992 339(8802):1138 1139 1349369 10.1016/0140-6736(92)90734-k

[23] Lindsay E A Chromosomal microdeletions: dissecting del22q11 syndrome Nat Rev Genet 2001 2 11 858 868 11715041 10.1038/35098574

[24] Yamagishi H Garg V Matsuoka R Thomas T Srivastava D A molecular pathway revealing a genetic basis for human cardiac and craniofacial defects Science 1999 283(5405):1158 1161 10024240 10.1126/science.283.5405.1158

[25] McQuade L Christodoulou J Budarf M Patient with a 22q11.2 deletion with no overlap of the minimal DiGeorge syndrome critical region (MDGCR) Am J Med Genet 1999 86 01 27 33 10440825

[26] Saitta S C McGrath J M Mensch H Shaikh T H Zackai E H Emanuel B SA A 22q11.2 deletion that excludes UFD1L and CDC45L in a patient with conotruncal and craniofacial defects Am J Hum Genet 1999 65 02 562 566 10417299 10.1086/302514 PMC1377955

[27] Sutherland H F Wadey R McKie J M Identification of a novel transcript disrupted by a balanced translocation associated with DiGeorge syndrome Am J Hum Genet 1996 59 01 23 31 8659529 PMC1915101

[28] Rauch A Pfeiffer R A Leipold G Singer H Tigges M Hofbeck M A novel 22q11.2 microdeletion in DiGeorge syndrome Am J Hum Genet 1999 64 02 659 666 9973528 10.1086/302235 PMC1377781

[29] Dallapiccola B Pizzuti A Novelli G How many breaks do we need to CATCH on 22q11? Am J Hum Genet 1996 59 01 7 11 8659546 PMC1915098

[30] Hall J G CATCH 22 J Med Genet 1993 30 10 801 802 8230153 10.1136/jmg.30.10.801 PMC1016557

[31] Kurnit D M Layton W M Matthysse S Genetics, chance, and morphogenesis Am J Hum Genet 1987 41 06 979 995 3687945 PMC1684364

[32] Amati F Conti E Novelli A Atypical deletions suggest five 22q11.2 critical regions related to the DiGeorge/velo-cardio-facial syndrome Eur J Hum Genet 1999 7 08 903 909 10602366 10.1038/sj.ejhg.5200399

[33] Digilio M C Marino B Capolino R Familial recurrence of nonsyndromic congenital heart defects in first degree relatives of patients with deletion 22q11.2 Am J Med Genet A 2005 134A 02 158 164 15669097 10.1002/ajmg.a.30587

[34] Heine-Suñer D Armengol L Torres-Juan L Clinical and molecular characterization of deletions, duplications and mutations in the 22q11.2 region[abstract]Eur J Hum Genet 2008 16 02 x

[35] Guris D L Fantes J Tara D Druker B J Imamoto A Mice lacking the homologue of the human 22q11.2 gene CRKL phenocopy neurocristopathies of DiGeorge syndrome Nat Genet 2001 27 03 293 298 11242111 10.1038/85855

[36] Vorstman J AS Chow E W Ophoff R A Association of the PIK4CA schizophrenia-susceptibility gene in adults with the 22q11.2 deletion syndrome Am J Med Genet B Neuropsychiatr Genet 2009 150B 03 430 433 18646052 10.1002/ajmg.b.30827 PMC3127866

[37] Guy C Wang X Lu X Lu J Li S Two patients with small chromosome 22q11.21 alterations and central nervous system abnormalities Mol Cytogenet 2015 8 102 26719767 10.1186/s13039-015-0200-1 PMC4696335

[38] Firth H V 22q11.2 duplication – retired chapter, for historical reference only Seattle University of Washington 2009 20301749

[39] Precht K S Lese C M Spiro R P Two 22q telomere deletions serendipitously detected by FISH J Med Genet 1998 35 11 939 942 9832042 10.1136/jmg.35.11.939 PMC1051488

[40] Durand C M Betancur C Boeckers T M Mutations in the gene encoding the synaptic scaffolding protein SHANK3 are associated with autism spectrum disorders Nat Genet 2007 39 01 25 27 17173049 10.1038/ng1933 PMC2082049

[41] Prasad C Prasad A N Chodirker B N Genetic evaluation of pervasive developmental disorders: the terminal 22q13 deletion syndrome may represent a recognizable phenotype Clin Genet 2000 57 02 103 109 10735630 10.1034/j.1399-0004.2000.570203.x

[42] Han K Holder J L Jr Schaaf C P SHANK3 overexpression causes manic-like behaviour with unique pharmacogenetic properties Nature 2013 503(7474):72 77 24153177 10.1038/nature12630 PMC3923348

[43] Sarasua S M Dwivedi A Boccuto L 22q13.2q13.32 genomic regions associated with severity of speech delay, developmental delay, and physical features in Phelan-McDermid syndrome Genet Med 2014 16 04 318 328 24136618 10.1038/gim.2013.144

